# Managing laboratory automation

**DOI:** 10.1155/S1463924695000149

**Published:** 1995

**Authors:** Thomas J. Saboe

**Affiliations:** Rhône-Poulenc Rorer Inc. PO Box 1727 500 Virginia Drive Fort Washington PA 19034 USA

## Abstract

This paper discusses the process of managing automated systems through their life cycles within the quality-control (QC) laboratory environment. The focus is on the process of directing and managing the evolving automation of a laboratory; system examples are given. The author shows how both task and data systems have evolved, and how they interrelate. A BIG picture, or continuum view, is presented and some of the reasons for success or failure of the various examples cited are explored. Finally, some comments on future automation need are discussed.


Journal of Automatic Chemistry, Vol. 17, No. 3 (May–June 1995), pp. 83–88

### Managing laboratory automation

**Thomas J. Saboe**
*Rhône-Poulenc Rorer Inc., PO Box 1727, 500 Virginia Drive, Fort Washington, PA 19034, USA*

*This paper discusses the process of managing automated systems through their life cycles within the quality-control (QC) laboratory environment. The focus is on the process of directing and managing the evolving automation of a laboratory; system examples are given. The author shows how both task and data systems have evolved, and how they interrelate. A* BIG *picture, or continuum view, is presented and some of the reasons for success or failure of the various examples cited are explored. Finally, some comments on future automation needs are discussed.*

### Introduction

The laboratory automation process continues to evolve and is becoming increasingly sophisticated and complex. It is diverse—systems are seldom clones as dictated by the specific needs of various laboratories. It is interdependent—systems must interface with electronic and mechanical components, other systems, and with human beings. It will not go away—individual laboratory automation projects are only steps in a continuing process of renewal, growth, and integration. As laboratory managers we cannot ignore this process. We need to be aware of it and be able to work it to an advantage.

This manuscript discusses the management aspects of the laboratory automation process from the viewpoint of practical experience. The lessons of the last decade are offered as a guide or foundation. As we continue to pioneer laboratory automation we need to assimilate these lessons and build on them.

### Discussion

### *Why?*

One of the first questions managers must ask and be satisfied on is 'why automate lab systems and processes?'. The answer to this question sets the tone, the urgency and the value of the project. It also determines our own commitment level to the project, which is a key step in assuring its success. If convinced of the worthiness of the project, then we have gone a long way towards completing it.

The most obvious answer is corporate pressure to reduce costs. While always a key business strategy, the particular environment of the pharmaceutical industry over the last few years has dictated renewed emphasis on cost control and reduction. Many of us in this industry are now working with lower staff levels than ever before. Yet our workloads have remained the same or even increased. Automation offers a practical way out of this dilemma.

Another very strong incentive for laboratory automation is safety. Operations involving hazardous materials should be automated. Robots, or automatic dispensers, for example, provide means of performing tasks with minimal human exposure to the hazardous material or environment. Management has the responsibility to provide these alternatives when they exist and also the mandate to develop additional alternatives if at all possible.

Finally and not surprisingly, scientists share basic desires to improve, to learn and to advance technologically. Laboratory automation is a natural outgrowth of those tendencies. It provides a practical forum to be creative and pioneering. Managers need to recognize these qualities and encourage laboratory automation as a rewarding developmental path.

### *What?*

What is it that managers need to do regarding automation? Part of the answer is to plan. To do so requires an awareness of what is possible given the current state of technology. Since technology is always changing and vastly diverse, managers need to continually find creative ways to keep up. There is no set answer to the problem. A cartoon from some time ago aptly caught the spirit of the situation. It depicted a derelict (tramp) on a park bench and two passing scientists, one of whom was remarking to the effect that the derelict used to be a leader in his field, but could not keep up with the literature. Of course none of us have the time to really stay current ourselves, but we can use the eyes, ears and especially the minds of our colleagues. Their natural interest in specific problems will help focus on and shape the best solutions. All we need to do is pay attention.

### *When?*

Planning also requires a sense of timing, as well as a sense of the current status of any systems already in place. Systems follow a generalized life cycle—they are conceived and developed and then serve a useful purpose for a time. However, things change and systems need to change, grow or adapt as needs require, or be relegated to the scrap heap. Managers need to maintain an awareness of the life cycle stages of all the systems currently in place. This knowledge will help us to plan when we need to invest in adapting or integrating systems in light of our current needs and emerging technology. A practical example might be planning to upgrade a robotic or LIMS system well in advance of the termination of support for the current system. It sounds obvious, but failure to plan for this eventually for even the smallest subsystem can result in serious complications later.

© Zymark Corporation 1995

*Who?*

Managing human resources is an integral part of managing laboratory automation. Developing the level of competence an individual needs to put an automation project together takes a great deal of training and commitment. This can be particularly difficult if you manage a front door operation like those frequently found in quality control. That is, a unit with a high proportion of entry-level positions and a high turnover rate. If this is your situation, then select your candidates with care and get them involved in automation projects early in their careers. Look for developmental skills and knowledge, rather than just operational skills or experience. Problem solving, creativity and commitment level make good indicators.

Usually just being selected to work on a laboratory automation project motivates an individual. It is something new and different. The real problem managers face is keeping that person motivated throughout the development stage, which in some instances can run for extended periods of time. It can also become extremely difficult should the project hit a serious problem. Managers need to know the individual and provide whatever support they can during this time. Listening to the individual and providing guidance are good, proven first steps. Exercise direction and/or support as required.

Knowing the needs and aspirations of the individual will determine the best way to reward the individual for their involvement in the project. Internal intradepartmental recognition of efforts is often helpful in keeping a person motivated during a project. It is usually better to save internal interdepartmental or company wide recognition for the completion or operational stage of the project. On one project, which will be examined later, the idea of publishing the project in a well-known trade journal held high value in the minds of the project team members, and helped keep interest and motivation high through some trying times. Other individuals shun the limelight and find satisfaction just in exploring new technology. Exercise caution in directly linking increased compensation or advancement to laboratory automation projects. While such projects correctly enhance an individual's career, never forget that his or her associates had to cover that person's routine work in order to allow the time to devote to the project. Stress the accomplishments of the benefits to the entire laboratory staff.

*How? A prescription for success*

Laboratory managers need to bring all of the above together to ensure the success of the automation project. Resources such as personnel, financial, time, and technology need to be assessed and redistributed as best possible.

An essential step in ensuring success is to choose a champion for the project. This person absolutely must believe in the project and be willing to commit to seeing it through. Depending on the project, the champion may operate alone or head up a team. It does not matter, provided the person has the appropriate technical *competence*, temperament and if necessary the team skills to function in the pertinent role. What does matter is that he or she takes up the cause. This person should be someone at the technical level, not managerial, simply because most managers do not have the luxury of focusing entirely on one system or project for great lengths of time. The champion must have the *vision* to see the benefits the assignment will bring to the organization. But, knowing where to go is not enough. This person must also have *leadership* skills to be able to bring the rest of the staff and all support personnel along to the new operational destination. As a side note, the manager should prepare the champion to deal with the 'It'll never work' crowd. Resistance to change and criticism are more than likely to crop up and the champion can and should deal most effectively with them. Finally, the person must take responsibility for and *ownership* of the project or system for the duration of its development. Both the manager and the individual should commit to the project. This ensures that the individual is not constantly wondering if he or she will be taken off the assignment, and also allows the manager to take a step back away from it.

Big projects may exceed personnel or technical resources. In these instances outside help is an excellent way to bring the system into reality. Using outside help is effective resource management. Two big advantages are that (1) it avoids hiring permanent people; and (2) it can often speed up the project technically. Financially this makes good sense. This approach works effectively in a team environment. Such a scenario might have several people from each firm meeting regularly; to solve problems, issue status updates or refocus on objectives. The contractor or vendor can often bring in expert mechanical, electronic or computer resources that are beyond the scope of your company's expertise. On the other hand, your company can usually supply the scientific knowledge, support, and testing facilities that are necessary. The contractor or vendor also may have had to deal with similar problems with other clients and without compromising their confidentiality agreements, suggest alternative avenues of attack.

Should you decide to use an outside contractor, or work with an instrument or equipment vendor, look for one that is willing to work with you over the long term. Ask for evidence of successful applications that they have installed and have operating at other companies. If possible, it is worth the phone call or visit to see how satisfied the customers really are. Plan to develop a long term 'partnering' relationship with the firm. Doing so opens up better communications for the initial project. Consider that if the outside agent knows what is being planned for the laboratory's automation future, then compatibility can be built into the various individual systems. Later, when it is time to upgrade or integrate systems this may pay off in terms of easing the interfacing or communications problem.

Managers must gauge the level of corporate commitment before embarking on a major automation project. A company that puts up initial capital, but then is unwilling to finance yearly service and maintenance agreements or provide the required support facilities and personnel training is lacking in commitment. Automated systems are sometimes subject to failure just as humans are sometimes prone to illness, and both can result in decreased productivity. Managers need to appraise the level of support each system requires in relation to its impact on operations should it fail. The corporation should accept that assessment and provide for it. Other important considerations for the automation manager are the corporate willingness to set aside the time to complete the project, and the corporate acceptance of or belief level in automation in general and the project in particular. In general, know your environment. Is it conducive to automation, just tolerant, or are the odds stacked against you?

Managers being aware of what technology is possible, must then examine what is available and at what cost. Just because a new gadget has been developed does not guarantee that it is the right gadget or most cost effective for your purpose. We have all heard the term 'vaporware'. Be sure that the object or software actually exists, is in production and being supported before purchasing it, if at all possible. Most systems we are developing are unique enough in their purposes without building in additional hard to replace parts or software. Weigh the risks before committing your resources. We learned this lesson the hard way when a board in a specialized autotitrator needed replacement. We discovered that the initial customization necessary in that early application had not been documented by the vendor or by the company. Furthermore, the expert technician had left the vendor's company. The vendor was then unwilling or unable to decipher and replicate the electronics, leaving the application permanently disabled. Although this lesson predates the pharmaceutical industry's renewed focus on validation, it certainly underscores the importance of that necessity as well.

Have a development plan. It is very helpful to have the project goals, purpose, resource allocations and other facts laid out in an orderly fashion. It should begin with a requirements analysis. That is, what is it that we expect the completed system to be able to do. Focus on the essentials, and state them in clear language that everyone agrees on. This step can be invaluable in keeping you and your associates focused and on track through the developmental stages.

Set the milestones, and determine what constitutes meaningful measurements and the acceptance criteria. If possible, outline the validation protocol at this stage. It is also the time to discuss how to fit the new system in with existing old systems and plan ahead for integration with future systems. For example, if you are adding another loop to your existing chromatographic integration system, consider how it will affect the overall performance of the whole system.

Historically, most of our laboratory automation projects were solutions to existing QC laboratory bottlenecks or problem areas. This is a perfectly fine starting place for many projects, but it should really begin back at the analytical development stage. Analytical methods need to be developed with automation in mind. Too often methods arrive in QC labs totally geared to manual execution. Encouragingly, there are many signs that this is changing. However, we as end users and managers must continue to challenge our methods development and research colleagues to design analytical methods that are compatible with automatic analysis techniques and processes. A codevelopment process involving the end user group similar to that outlined above for the third party contractor might offer a possible solution. Another alternative might be to have the end user group contribute to or review the requirements analysis package before starting the project.

In the past, laboratory automation projects were often viewed as simple trips going from here to there in the technological sense. The project ended when we arrived at our new technological solution. A kind of 'Let's motorize that operation' or 'Let's teach a robot to sample dissolution vessels, and we can go do something else' mentality. More recently a somewhat different picture of laboratory automation has emerged. In this model we never really arrive at a permanent destination. In it, we recognize that we are constantly rebuilding the vessel which allows us to go from here to there, and that this process is at least equally important to the fact of arrival. We started with very simple systems, a rowing boat, for example, and by innovating, and linking and redesigning and working together we begin to see the outline of a modern cruiser. These qualities are what make automation attractive and rewarding to individuals, while the financial and economic end results satisfy the corporation. It is our job as managers to recognize these qualities and assemble all of the components into a final symbiotic unity. When we achieve this the project will be successful.

### Examples

### *Prehistory*

Probably one of the simplest examples of laboratory automation is the magnetic stirrer. Not very impressive to look at, but it certainly made life much simpler for those of us lacking in eye-hand co-ordination while titrating. On the lab automation evolutionary scale its use of pure electro-mechanical and magnetic components makes it the rough equivalent of the amoeba. Other early examples might be auto injectors or auto samplers. Again, fantastic labour saving devices for those previously accustomed to late nights spent making chromatographic injections every few minutes. These devices made evolutionary progress by utilizing elementary electronic brains. Another development was the early electronic integrators. A case of even higher level electronic brain power as opposed to mechanical muscle.

### *Dissolution*

Our first robotic application was a semiautomated dissolution system [1]. The application was for a timed release product which required periodic sampling over a 10 hour run. From an evolutionary standpoint, our automated system had acquired mobility and grasp. In a sense we were beginning to climb out of the swamp. This application went on-line in August 1983 and is still in operation today. While consisting of relatively simple sample removal and pH adjustment operations, it loosened many of the cords tying the analyst to the job. The analyst could not just walk away and forget it for the duration, but he or she could now keep an eye on it from afar. Success in this endeavour can be credited to the foresightedness of the managers, the simplicity and limited scope of the requirements, the stubbornness and enthusiasm of its champion and its high payback rate. This initial application taught us much about the care and feeding of more complex automatic systems and their limitations. Part of the operation requires the use of disposable filter tips which tax the limits of the robot's positioning system in order to pick them up. We went through several redevelopment cycles trying to improve the robot tip accuracy and reliability. Current efforts to resolve this problem include using a permanent embedded filter that can be cleaned in place. The core lessons: (1) explore what appears to be limiting factors early and thoroughly in the process before investing heavily in the system; (2) find your champion and begin filling in his or her developmental needs.

The next step in our development of the dissolution system was to automate the data collection and calculation end. Our initial system was several stand-alone microcomputers hooked to individual UV/Vis spectrophotometers serving set dissolution baths. This advance brought significantly more computing power to bear, thus eliminating the reading of peak heights from the strip chart recording and the manual calculating of results. An in-house algorithm was developed to smooth the data and pick the best point to read the peak height. Generating a final report manually used to take about 6 h per dissolution run. The computer system cut that down to about 6 min. This system was very successful. One advantage was that it eliminated variability in the way peaks were read. Another was its versatility. We could use the program for many different products and dosage strengths. In fact we modified the program to accept a signal from a manually operated probe that an analyst could use when running manual dissolution. Yet another advantage was that the calculations were now validated, giving us greater confidence in our results and reducing our checking time. However, when the computer manufacturer unexpectedly went out of business, it became virtually impossible to maintain the equipment. The lessons were: (1) that there are big payoffs in automating data handling; (2) weigh the probability of future vendor support carefully during the risk analysis stage of the project.

Our second development cycle was also successful, and is what we are currently using. This development project was the first time we used a third-party developer to actually perform the work. The computers mentioned above were replaced with PCs in a local area network configuration. This system is much more user friendly, performs the same data handling, and also brings a fair dose of data backup and error handling abilities into the system. The key lessons from this cycle were that third party developers and codevelopment teams were cost effective and efficient resource options. It also was the first time we relied on a true development plan (at the insistence of the developer) which repeatedly brought us back to the original intent and scope of the project. We remained on track and focused. Another innovation was designing the system to be configurable. This approach enables the system to be redirected time and again for different applications without opening up the entire system. It also allows supplemental validation rather than comprehensive as new products are brought on line.

### *LIMS*

Probably one of the most complex, costly and yet interesting and effective systems a manager will run across is a LIMS or Laboratory Information Management System. These systems were developed over the last decade and represent a significant advance in laboratory data management. There are three main functions which make a LIMS a virtual necessity in any large laboratory. First, calculations can be programmed and validated in a LIMS making data crunching easier for your analysts and giving the manager better regulatory control. Second, historical data can be called up and utilized very quickly. Making informed decisions thus becomes easier and more timely. Data that previously had to be gathered, sorted, crunched and plotted manually can be had in minute fractions of the time. LIMS put more data into the hands of more laboratory people, who could then turn it into information, than ever before. Finally, LIMS gives management and staff a window to preview, organize, review and manage the workload. What is due today or next week can be asked and reasonably answered without spending unreasonable time or resources finding out.

A LIMS is subject to change, revision and ultimate obsolescence just as any other system. In our case, the LIMS vendor withdrew from the business forcing us to address replacing our LIMS. Even if this had not happened, we were outstripping the capabilities of our original LIMS, and already planning to replace it. In doing so, we have asked ourselves what did we learn in implementing and using the first system and what do we need in our next system. One lesson is that the system will not remain static. The planned users and uses will grow as more and more people in the company realize the benefits of having access to the laboratory data. For example, it was originally expected that analysts would only enter laboratory data. But, as they gained experience, confidence and responsibility, they needed access to more data and more functions to help make better decisions. The functions and tools for reporting that data had to be provided to them. Managers need to keep reassessing who can and should be doing what with the data.

Our paradigms must change with each new LIMS system. Both LIMS implementations required rethinking how we do business. Questions such as 'Do we need Analytical Report Forms?' or 'Can the analysts use the terminals to display work schedules?' are examples. Frequently the new system is unable to display information in the same format that we are accustomed to. The manager's task is to ensure that the users get the training they need to enable them to find the necessary information. This in turn assures the system will reach its productivity goals as quickly as possible. In our laboratories, we are using a phased approach. We have our personnel divided across four product-focused teams, and we are trying to phase in LIMS one team at a time. This keeps the training classes relatively small. It also divides the workload up in a fashion that addresses the major products and components of each team first, instead of trying to bring everyone and everything up at the same time. This keeps the workload focused and manageable for those configuring or learning the system.

This project uses most of the ideas for success as outlined above. The project has a dedicated champion. Besides being convinced of the benefits the new system, the project manager had the additional incentives of being responsible for a major project and the opportunity to demonstrate his financial management and leadership abilities. A team approach and a good relationship with the new LIMS vendor characterized the project. Significant time and thought went into the requirements analysis and the selection of the vendor. Corporate commitment was obtained early and remains high. The value and necessity of the first LIMS was one selling point. Another important reason was the similarity and connectivity of the system with other corporate systems. Our new LIMS will connect with our chromatography system and our inventory system. Ultimately, information from our site can be shared with other worldwide sites as needed.

The still dawning realization, by managers, that laboratory systems cannot operate in a vacuum and need linkages to our inventory systems, instruments and our worldwide plant and research facilities deserves credit, attention and continued cultivation. For automatic systems to be truly useful they must be able to accept, process and send data to and from other systems. Various pharmaceutical operations use different pieces of information about products, raw materials or packaging components. One group may need the lot number, another the location of the material. Most of today's systems are designed to capture and use only part of the information relating to the material. While it would be impractical to build one big system to handle every minute detail, the next best thing would be to make the data in one system easily transferable to another. Imagine a QC manager being able to electronically link vendor lot numbers, suppliers, delivery information or whatever from the inventory system, combine it with his or her laboratory data, and send it off to, for example, a director on the other side of the world.

It could save tremendous time and effort, and lead to even better quality decisions.

*Recycling*

Some years ago we attempted to automate a liquid-liquid extraction procedure for Simethicone, a defoaming agent used in antacid products. A robotic system was developed, validated and placed into service. However, it did not last long. The main problem was its lack of reliability. The robot was required to uncap and cap 50 mL disposable screw cap test tubes. Variations in the disposable test tubes and the complexities of screwing a cap on a bottle robotically were the root causes. The caps had to be tight enough to prevent leakage and evaporation and loose enough for the robot to grip and remove them. The lesson we learned was that we had not adequately assessed the current level of robot technology in view of the operations we expected it to perform. Perhaps snap or crimp caps would have been better alternatives.

The robot from the simethicone application was redirected to perform simple dispensing operations for the analysis of solid dose antacid products. The fact that the same robot could be reapplied to a different problem was a big advantage. This time, the robot simply added a measured amount of acid solution to empty test tubes arranged in a rack. An analyst did the capping, pipetting and other operations: it was basically an early version of the modern Zymark BenchMate. Although it underutilized the robot's capabilities, the system was very efficient. With it, an analyst could prepare 100 to 150 unit dose samples per day. These abilities could be major advantages when you have changing needs or a temporary situation involving a high volume of routine sample preparation work. Be careful, however. Such work can be tedious for many individuals. If the assignment is going to run long term it might be better to invest in a completely automated solution.

Finally, reassigned to a third project, this robot is testing metered dose inhalers (MDIs) [2]. This application represents near state-of-the-art in laboratory automation. It combines a robot component, specialized mechanical actuators, a configurable program running on a PC controlling the events, ties to a local area network and instrumentation. This is by far the most complex project we have attempted. The core of the system is its ability to automatically actuate the MDI valves. The spray can be either wasted or collected and measured. The system frees the analyst from the tedium of manually actuating the valve hundreds of times per sample. Its development however, was delayed by resource shortages, primarily personnel, by technological problems and by a natural catastrophe. Personnel turnovers caused a succession of champions. Each new champion had a learning curve to ascend which slowed progress. Each departure of a champion took some knowledge away from the project, sometimes causing some lessons to be repeated the hard way. Managers should always be looking for and cultivating the understudy. However, this case exemplifies what can happen when either you do not have a replacement person in mind or when the turnover rate does not allow adequate training of the junior partner. Technology wise, unexpected problems arose with the original tubing used to plumb the unit. Although the vendor had published that the material was compatible with methanol it leached something that not only coated our flow cell's internal surfaces but interfered with the UV assay. Finally, mother nature dealt a severe blow at a critical point just after the initial installation. A lightning strike blew some of the major electronic components, illustrating the wisdom of investing in power surge protectors and uninterruptible power supplies at the very beginning of the project. Purchase them as part of the capital if possible.

Despite these problems, the project is nearing completion. It currently is validated for two of the three products we planned to run on it. The third product looks very close. The use of a third party codeveloper on this project was very successful. The codeveloper brought in some of the same individuals who developed our dissolution data collection system. As a result, we realized some of the benefits of our 'partnering' relationship with the developer. The new program used some of the programming ideas and even some code from the prior project.

The designers linked the system to our laboratory dissolution LAN which will ultimately be connected to our new LIMS. When finished, the system will allow samples to be prepared, tested and the data passed up to LIMS with minimal human intervention. Also, the specialized mechanical expertise and computer programming brought in by the codeveloper far exceeded our own limited resources. The team, despite the personnel changes, worked well. It actually helped bring new people up to date faster than if they had been working alone. Ideas were discussed openly and scientifically. It was a forum in which ideas could be tossed around, reassembled, re-examined and molded. These aspects were critical to our screening study on selecting the best method of collecting the aerosol dose as well as in solving nearly all of the problems which arose.

*Autotitrators*

Our first experience with autotitrators did not work out as well as we had hoped. The system performed an automated Acid Neutralizing Capacity (ANC) test. This was the application mentioned earlier that required the specialized electronics that the supplier could not resupply.

Our second attempt used off-the-shelf products and was very successful. This time, the task was to automate the $Al(OH)_3$ and $Mg(OH)_2$ assays and content uniformity tests on some antacid tablets. Prior attempts at some of our sister facilities to automate these tests had failed. Primarily, because the systems they tried used very different chemical properties to determine the endpoints. We decided to keep it simple. We stayed with the simplest, proven chemical technique, EDTA type titrations, and used a fibre-optic probe and a colorimeter to sense the endpoints. There were two unexpected technological problems which we had to overcome. The first was bubbles in the solution being titrated. In manual titrations small bubbles are of no consequence to the eye of the analyst, but even one bubble passing through the light path of a small probe can trigger a false endpoint. The solution to the problem uses an ultrasonic probe at a pretitration station to degas the solution. The second major problem was compatibility of the fiber optic material with the solvent. A Lexan sheath solved that problem. From a management perspective, this project was interesting because a suggestion to attempt to publish the project was taken taken to heart by the individuals developing the system. It was remarkable how much of an incentive this seemed to be, and how much pride they took in the finished article [3]. This system is still in use today. Also, it has been used by our methods development people to develop new analytical methods and to assist in several process validation studies.

### Summary

Managing laboratory automation is a complex job. All of a manager's skills are necessary. The automation manager must use resource management, perspective, scheduling and planning as well as technical and interpersonal skills. Given the present business and technological environment, these managers must maintain a higher level of technical awareness and expertise than ever before.

The job requires attention to past, present and future systems as well as personnel development. There is a cyclic pattern in a manager's activities, where he or she has to scale the heights, check the position and navigation of all their people and systems, and then slide down again into the details of each person and system in order to bring them along. The job is further complicated by the uniqueness of each and the ultimate need to integrate them all into one vast interacting system capable of operating and communicating on a global scale. It can be frustrating at times, but when you watch one of your people show off his or her project to the world, you can take deep, personal pride and satisfaction in a job well done.

### Acknowledgements

The author would like to thank the following for their contributions to laboratory automation: Dr J. N. Little, B. Lightbody, *et al.* of Zymark Corporation for their help and expertise with all of our varied robotic applications over the last decade; Mr J. Waters, *et al.* of InnovaSystems Inc. for their help and expertise with our computer and dose delivery systems; Mr T. F. Dolan of Rhône-Poulenc Rorer Inc. for his help and guidance on all our automation projects; and, finally, all the champions and technicians of Rhône-Poulenc Rorer Inc. who worked on all our projects.

### References

1. Dolan, T. F., Saboe, T. J. and Sattler, L. M., In *Proceedings of the 131st American Pharmaceutical Association Annual Meeting*, Montreal, Canada (May 1984).
2. Finley, T. A., Landes, N. A., Fuchs, R. M. and Zepka, A. J., In *Proceedings of the International Symposium on Laboratory Automation and Robotics* (1993), p. 163.
3. Spatola, D. C. and Warfield, S. J., *American Laboratory* (May 1993), p. 66.

